# Conversion surgery vs. standard of care in pancreaTic cancer oligometastatic to the liver (SONAR: Surgery in Oligometastatic paNcreatic cAnceR) a randomized controlled trial

**DOI:** 10.1186/s12885-025-15183-9

**Published:** 2026-01-12

**Authors:** Riccardo Guastella, Giampaolo Perri, Domenico Bassi, Nicola Canitano, Virginia Padoan, Samuele Grandi, Riccardo Pellegrini, Letizia Procaccio, Francesca Bergamo, Sara Lonardi, Riccardo Carandina, Enrico Gringeri, Giovanni Marchegiani, Umberto Cillo

**Affiliations:** 1 University of Padua, Department of Surgical, Oncological and Gastroenterological Sciences, Padua, Italy; 2 Hepato-pancreato-biliary and Liver Transplant Surgery Unit, Padua University Hospital, Padua, Italy; 3 Medical Oncology 1, Veneto Institute of Oncology IOV-IRCCS, Padua, Italy; 4 Radiodiagnostic Unit, Padua University Hospital, Padua, Italy

**Keywords:** Pancreatic cancer metastatic to liver, Pancreatic cancer oligometastatic to liver, Conversion surgery, Pancreatic cancer, Neoadjuvant therapy for oligometastatic pancreatic cancer, Resectable pancreatic cancer, Resectable liver metastasis in pancreatic cancer, Combined surgery for metastatic pancreatic cancer.

## Abstract

**Background:**

Pancreatic cancer oligometastatic to the liver represents a distinct subset of advanced disease, presenting a limited number of metastases in a single site. First-line chemotherapy is considered the standard of care, with a poor overall prognosis. However, the optimal strategy for oligometastatic patients presenting response or stability after treatment is unclear. In selected patients, surgical resection is associated with prolonged survival, according to retrospective series. The aim of this randomized clinical trial is to compare the efficacy and safety of surgery versus observation or continuation of chemotherapy in patients with resectable pancreatic cancer oligometastatic to the liver with stable disease or response after first-line chemotherapy.

**Methods:**

The study is a phase-2 multicentric randomized controlled trial with 1:1 allocation ratio. Patients diagnosed with pancreatic cancer and a limited number (up to 3) of liver metastases, with no evidence of extrahepatic disease, who received systemic chemotherapy as the initial treatment and with disease response or stability after therapy will be considered eligible patients. Patients will be randomized to either Arm A (surgery) or Arm B (observation or continuation of chemotherapy). The primary outcome will be overall survival at two years, with secondary outcomes including progression-free survival, treatment-related adverse events, quality of life and translational analyses.

**Discussion:**

This randomized controlled trial will evaluate the role of surgery in pancreatic cancer oligometastatic to the liver after response or stability to first-line chemotherapy. While systemic therapy remains the standard of care, selected patients may benefit from surgical resection. By comparing surgery to observation or continuation of chemotherapy, the SONAR trial aims to fill a critical gap in treatment strategies and potentially refine the management of this challenging disease.

**Trial registration:**

The trial has been registered at ClinicalTrials.gov on 15/11/2024 before inclusion of the first patient (NCT06690528).

## Background

Pancreatic cancer represents a highly aggressive disease with a dismal prognosis, ranking as the fourth leading cause of cancer-related deaths worldwide [1, 2]. Metastatic pancreatic cancer is characterized by the dissemination of tumor cells from the primary site to distant organs through the lymphatic or blood circulation. At diagnosis, most patients present with either locally advanced (30–35%) or metastatic disease (50–55%)[3]. In these settings, surgery is generally not indicated due to the extent of disease, although highly selected patients with favorable biology and response to systemic therapy may be considered as surgical candidates at high-volume centers [4]. Among the various sites of metastasis, liver involvement is particularly common and associated with a poor outcome [5]. The liver contributes to the significant morbidity and mortality observed in patients with metastatic pancreatic cancer and the presence of liver metastases is associated with a poorer prognosis, decreased overall survival (OS), and limited treatment options [5, 6]. Despite improvements in diagnostic techniques, treatment modalities and supportive care, the prognosis for patients with metastatic pancreatic cancer remains discouraging. The median survival is typically less than one year, and the five-year survival rate is less than 5%[2, 3]. The management of metastatic pancreatic cancer poses significant clinical challenges. The presence of distant metastases implies a disease aggressiveness nullifying the effectiveness of localized treatments, such as surgical resection, which is the treatment of choice in localized pancreatic cancer [4]. The conventional approach for metastatic disease is therefore systemic chemotherapy, aimed at controlling tumor growth, reducing symptoms, and improving quality of life (QoL). The limited efficacy of conventional systemic chemotherapy regimens in terms of OS underscores the urgent need for novel therapeutic strategies. The role of aggressive local therapies, such as surgical resection of the primary tumor and liver metastases, is an area of active investigation and clinical debate for those patients who show disease response or stability after treatment in the context of oligometastatic disease.

Oligometastatic stage, defined as the presence of a limited number of metastatic lesions in specific organs, offers a potential opportunity for locoregional interventions. Currently, there is a lack of consensus and of robust evidence regarding the optimal treatment approach for patients with oligometastatic pancreatic cancer to the liver, and patients with hepatic oligometastatic adenocarcinoma of the pancreas receive palliative treatment only. The concept of oligometastatic disease challenges the traditional notion of systemic disease spread and suggests that a subset of carefully selected patients may benefit from aggressive local therapies targeting the metastatic sites [7–9]. In this context, surgical resection of both the primary tumor and liver metastases in oligometastatic pancreatic cancer has gained increasing interest in selected patients. Proponents of surgical intervention argue that complete tumor removal after the delivery of optimal first-line systemic therapy may eradicate the primary source of cancer and eliminate or control the metastatic localizations. This approach could potentially improve disease control, survival, and QoL for selected patients. However, the available data on the efficacy and safety of surgery in the setting of pancreatic cancer oligometastatic to the liver are limited and primarily based on retrospective studies and small case series, with a clear selection bias towards the few patients harboring an extremely indolent disease biology after extended periods of systemic therapy and/or observation [10–18].

The lack of prospective randomized controlled trials evaluating the role of surgery in this specific patient population hinders evidence-based decision-making. Metastatic pancreatic cancer to the liver remains a clinical challenge with limited treatment options and a poor prognosis. The exploration of surgical resection as a potential therapeutic modality offers hope for improved outcomes in a subset of carefully selected patients. This study aims to compare the outcomes of surgical resection versus observation or continuation of chemotherapy in patients with resectable pancreatic cancer oligometastatic to the liver after response or stability at first-line chemotherapy. The findings of this study will provide valuable insights into the optimal management approach of this challenging subset of patients.

### Objectives and endpoints

#### Objectives

The primary objective is to assess the possible superiority of surgical resection of the primary tumor and liver metastases in patients with pancreatic cancer oligometastatic to the liver and response/stability after first-line chemotherapy, compared to observation or continuation of chemotherapy, aimed at prolonging survival and maintaining quality of life. Secondary objectives are to investigate efficacy and safety of this treatment strategy, and to identify biomarkers of response to oncologic/surgical treatment.

#### Endpoints

The primary endpoint will be OS rate at two years, defined as the percentage of patients alive at two years from treatment. Secondary endpoints include progression-free survival (PFS), treatment-related adverse events (AEs), surgical morbidity and mortality, QoL measurements using validated questionnaires like “European Organisation for Research and Treatment of Cancer Quality of Life Questionnaire-Core 30” (EORTC QLQ-C30) and the pancreatic cancer-specific module (EORTC QLQ-PAN26) [19, 20], and exploratory analyses of established and emerging prognostic factors including Carbohydrate Antigen 19 − 9 (Ca 19 − 9), ratio between neutrophils and lymphocytes (N/L ratio) and circulating tumor (ct) DNA.

### Study design

**Fig. 1:**
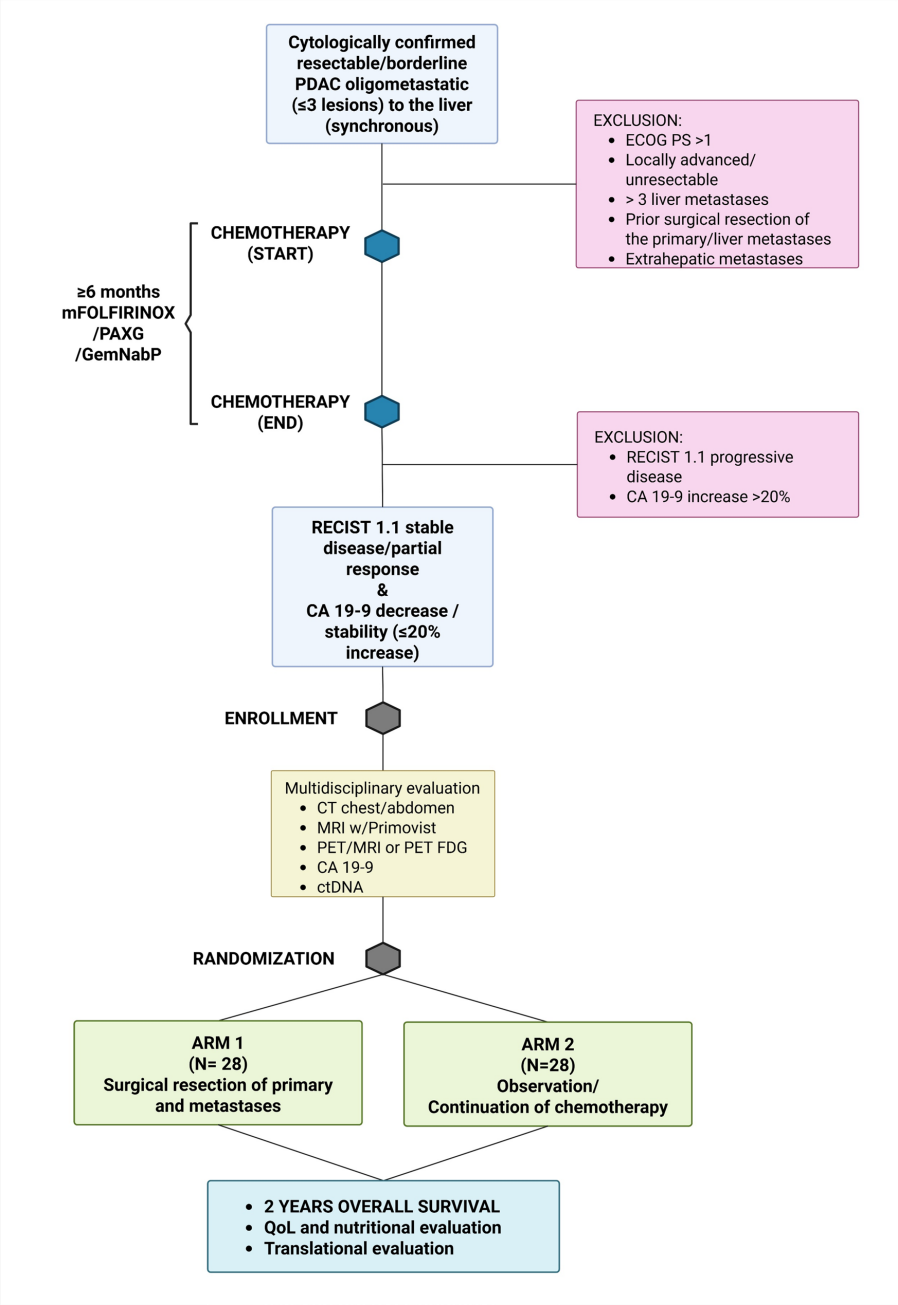
CONSORT compliant flowchart of the study protocol

This is a phase-2 multicenter randomized controlled trial with a 1:1 allocation ratio. Eligible patients with pancreatic cancer oligometastatic to the liver with response/stability after first-line chemotherapy will receive surgical resection or observation/continued chemotherapy. The study will be conducted at different international referral centers with high expertise in pancreatic cancer treatment to ensure the highest standards of quality and an adequate sample size. After the enrollment of the first 20 cases, an independent data monitoring committee (IDMC) will review safety and futility (using 90 days major morbidity + mortality as the endpoint), to allow for early study termination or modification if necessary. The study design is represented in Fig. 1.

### Study population

The study will enroll adult patients aged 18 years or older with cytologically or histologically confirmed pancreatic adenocarcinoma and radiologically documented liver metastases. Eligible patients must present with synchronous oligometastatic disease, defined as a limited number of liver metastases (up to 3 lesions) without evidence of extrahepatic metastases based on multidisciplinary discussion. The primary pancreatic tumor must still be in place at the time of enrollment. Enrolled patients must have received a minimum of 6 months of systemic chemotherapy. This requirement reflects current guideline-based practice, where approximately six months of first-line treatment is considered the standard duration when tolerated and in the absence of progression. Shorter induction periods may risk undertreating patients who could benefit from continued disease control; therefore, six months was chosen as a clinically meaningful threshold for trial eligibility. The systemic treatment will consist preferably in 12 cycles of modified (m)FOLFIRINOX administered every 2 weeks (oxaliplatin 85 mg/m^2^ day 1, irinotecan 150 mg/m^2^ day 1, 5-fluorouracil continuous IV infusion 2.4 g/m^2^ over 46 h, and leucovorin at a fixed dose of 400 mg/m^2^) or alternatively 6 cycles of PAXG administered every 4 weeks (cisplatin 30 mg/m^2^ every 2 weeks, nab-paclitaxel 150 mg/m^2^ every 2 weeks, capecitabine 1250 mg/m^2^/day for 28 consecutive days, and gemcitabine 800 mg/m^2^ every 2 weeks) as clinician’s choice based on patient’s performance status, comorbidities, detection of germline BRCA 1–2 pathogenic variants. Patients without disease progression, stable disease, or partial response after 6 months of first-line chemotherapy, as determined by RECIST 1.1 criteria [21], will be randomized for surgery or continued chemotherapy. Patients with unresectable disease, significant comorbidities, or contraindications to surgery will be excluded from the study. Patients needing palliative procedures (i.e., presenting with jaundice, gastric outlet obstruction) will be evaluated for inclusion only after palliation.

#### Inclusion criteria

- Adult patients aged ≥ 18 years and ≤ 75 years (at diagnosis).
- Cytologically or histologically confirmed pancreatic adenocarcinoma either resectable or borderline resectable (at diagnosis) according to National Comprehensive Cancer Network (NCCN) [4] (see Sect. 5).
- Synchronous oligometastatic disease (at diagnosis), defined as a limited number of radiologically documented liver metastases (up to 3 lesions).
- No evidence of extrahepatic metastases (at diagnosis).
- Eastern Cooperative Oncology Group (ECOG) performance status (PS) 0–1 (at enrollment).
- Partial response or stable disease after completion of first-line chemotherapy, as determined by RECIST 1.1 criteria [21] (modified to exclude any % of increase in the sum of diameters of target lesions) (at enrollment).
- Decreasing or stable (defined as ≤ 20% increase) serum CA19-9 level after chemotherapy (at enrollment).
- Liver metastases considered resectable (see Sect. 5) or alternatively treatable by needle ablation/microwave once no larger than 20 mm (at enrollment). Eligibility of liver metastases will be determined by multidisciplinary tumor board discussion at participating centers, rather than predefined dimensional thresholds.

#### Exclusion criteria

- Locally advanced pancreatic cancer according to NCCN [4].
- Unresectable liver disease (according to multidisciplinary discussion).
- Involvement of other organs.
- Presence of significant comorbidities precluding surgery.
- Pregnancy.
- Contraindications to surgical resection.
- Prior surgical resection of the primary tumor or liver metastases.
- Evidence of extrahepatic metastases.
- Inability to provide informed consent or participate in follow-up assessments.
- Disease progression as determined by RECIST 1.1 criteria [21] (modified to include any % of increase in the sum of diameters of target lesions) after chemotherapy.
- Serum CA19-9 level increase >20% after chemotherapy.

Note: Additional specific exclusion criteria may be defined at each participating center based on their institutional guidelines and patient population.

### Multidisciplinary evaluation

A multidisciplinary evaluation will be performed at the time of enrollment (within 3 weeks of last chemotherapy cycle administration). The evaluation will include the following tests:

- Staging thorax + abdomen CT scan (with pancreatic protocol).
- MRI liver imaging with gadoxetate disodium (e.g., Primovist™) as contrast agent.
- PET/MRI or PET FDG, as per institutional clinical practice.
- Serum CA 19 − 9 level.

### Definitions of resectability

#### Pancreas

The pancreatic primary will be considered eligible to surgery if either resectable or borderline resectable according to the updated National Comprehensive Cancer Network (NCCN) recommendations [4].

#### Liver

Liver metastases will be considered eligible for surgery if their complete excision will allow preservation of adequate liver function, either through anatomical or non-anatomical resections. If radical resection is not feasible, ablation will be allowed for lesions < 20 mm that are technically suitable (i.e., not adjacent to the hilar vascular/biliary bifurcation). Final eligibility and treatment strategy will be determined by a multidisciplinary tumor board at participating centers.

### Treatment regimens

All patients enrolled in the study must have received at least six months of systemic chemotherapy, consisting in 12 cycles of mFOLFIRINOX or alternatively 6 cycles of PAXG, as the initial treatment. Given possible differences in clinical practice at participating centers, gemcitabine + nab-paclitaxel (GemNabP) is accepted as a chemotherapy regimen alternative to PAXG, and standard FOLFIRINOX alternatively to mFOLFIRINOX.

### Interventions

After completion of 6 months of first-line chemotherapy, eligible patients will proceed to the randomization phase, within maximum 3 weeks since the completion of multidisciplinary evaluation. Patients will be assigned to one of the two study arms:

- Surgical Resection Arm: (Experimental group) patients in this arm will undergo surgical resection of both the primary tumor and liver metastases. The surgical approach, extent of resection, and perioperative management will follow the standard protocols at each participating center. Venous vascular resections might be performed to reach radicality. Either standard or parenchyma-sparing liver resections might be performed for resection of the liver metastases. Alternatively, needle ablation/microwave on the liver lesions is possible for lesions < 20 mm if technically feasible. Postoperative chemotherapy and/or radiotherapy could be administered as per multidisciplinary decision based on case-by-case evaluation. Ablation will be considered only when radical resection is not technically feasible, as determined by multidisciplinary review.
- Observation or continuation of chemotherapy Arm: Patients in this arm will continue to be treated following standard of care, consisting in observation or continuation of chemotherapy according to investigator’s choice and duration of first-line chemotherapy. If required, they will continue with systemic chemotherapy, as received during the initial treatment phase, with a chemotherapy protocol based on the Institution’s approved guidelines and adjusted as for clinician’s choice.

### Assessment of study endpoints

Patients will undergo regular assessments at predefined intervals throughout the study period. Assessments will include clinical evaluations, laboratory tests, radiological imaging (such as computed tomography scans of the abdomen and chest), QoL and nutritional assessments. The frequency of assessments may vary depending on the study phase but will generally occur every 8–12 weeks as per clinical practice. These assessments will provide data on the primary and secondary endpoints.

### Outcome measures

#### Primary endpoint

- Two-year OS (2y-OS) rate, defined as the percentage of patients alive after 2 years from randomization.

#### Secondary endpoints

- PFS, defined as the time between randomization and diagnosis of progressive disease.
- Surgical mortality and morbidity, graded according to Clavien-Dindo classification [22], at 90-days after surgery.
- QoL calculated using EORTC QLQ-C30 and QLQ-PAN26 [19, 20]
- Nutritional assessment
- Treatment-related AE.
- Analyses of prognostic factors including serum Ca 19-9 levels, N/L ratio, and translational analyses including ctDNA on plasma samples.

### Statistical analysis

Based on previous case series [18] and on the primary endpoint of 2y-OS from randomization, we calculated the following sample size: assuming a power of 80%, a one-sided alpha of 0.05, a p1 (% of alive at 2 year – control group) of 10% and p2 of 40% (% of alive at 2 year – study group), the sample size required to test superiority is 25 patients in the control group and 25 in the active group. Considering an expected dropout rate of 10%, the final sample size will be a total of 56 individuals (28 per group). Patients randomized to the surgical arm who are found unresectable at exploration will remain in the primary analysis according to the intention-to-treat principle. A modified ITT sensitivity analysis excluding patients who did not receive any study treatment will also be performed.

Statistical analyses will be conducted using appropriate methods based on the nature of the data and study objectives. Survival analyses, including Kaplan-Meier estimates and Cox proportional hazards models, will be used to evaluate the primary and secondary endpoints. Subgroup analyses based on relevant clinical and pathological factors will also be performed.

### Randomization and blinding

Patients who meet the eligibility criteria will be randomly assigned in a 1:1 ratio to either the surgical resection arm (*n* = 28) or the observation/continuation of chemotherapy arm (*n* = 28). Randomization will be performed centrally using a computer-generated randomization sequence. Stratification factors, such as ECOG PS and serum Ca 19 − 9 level at enrollment, location of pancreatic primary, and participating center, will be used to ensure balance between the two study arms. Due to the nature of the interventions, blinding of patients and treating physicians to treatment allocation is not feasible. However, outcome assessors and data analysts will be blinded to the treatment assignments.

### Quality of life and nutritional assessment

#### QoL

The assessment of QoL is an essential component of this study to comprehensively evaluate the impact of different treatment strategies on patients’ well-being. QoL will be assessed using validated cancer-specific and pancreas-specific questionnaires to capture various aspects of physical, emotional, and social functioning. The EORTC QLQ-C30 and QLQ-C30 PAN26 will be utilized as the primary instrument for QoL assessment [19, 20].

The questionnaires will be administered to patients at predefined time points throughout the study, usually during regular follow-up visits. Patients will be asked to rate the extent of their symptoms and the level of functioning on a scale ranging from 0 to 100, with higher scores indicating better functioning and higher symptom burden. The QoL assessments will provide valuable insights into the impact of different treatment modalities on patients’ daily lives, treatment-related side effects, and overall well-being. The collected QoL data will be analyzed descriptively, examining changes over time and differences between the study arms. Subgroup analyses may also be conducted based on relevant patient characteristics, such as age, sex, PS, and treatment response. The results will provide important information for clinicians and patients when making treatment decisions, as they will help to weigh the potential benefits and drawbacks of surgical resection versus continued chemotherapy on patients’ overall QoL.

#### Nutritional assessment

Nutritional counseling will consist of evaluation by dietician as per current clinical practice. A specific dietary program will be planned based on the anthropometric measurements, the calculated calorie-protein needs of patient and an accurate medical history carried out during the first visit. The dietary plan will include the intake of natural foods, the consistency of which will be adapted to the possible presence of dysphagia. During the planned accesses at oncological center, the dietician could identify the nutritional problems of patients if symptomatic, then suggest the necessary dietary strategies to deal with any reported difficulties.

### Translational research

Blood samples collected at predefined time points will be used for translational analysis, including molecular and immunological profiling and the identification of biomarkers, such as ctDNA, that may be predictive and/or prognostic for pancreatic ductal adenocarcinoma oligometastatic to liver. Samples may be also used to develop and validate propensity score, and to allow the generation of statistically meaningful biomarker data.

### Participating centers and funding

Participating centers will be selected among third-level referral centers for hepato-pancreato-biliary surgery which are currently already offering surgical resection as a therapeutic option to oligometastatic pancreatic cancer patients after chemotherapy.

As surgical resection after chemotherapy is already offered to oligometastatic patients at the promoting center (Padova), it will be considered standard clinical practice and won’t require funding dedicated to inpatient reimbursement. If inpatient reimbursement is required from participating centers, this won’t be covered by the promoting institution.

### Ethical considerations

This study will be conducted in compliance with ethical principles outlined in the Declaration of Helsinki and in accordance with local regulatory requirements. Institutional review board (IRB) approval will be obtained at each participating site. Informed consent will be obtained from all study participants before enrollment, ensuring their understanding of the study procedures, potential risks, and benefits.

### Data management and analysis

Data will be collected prospectively using standardized case report forms (CRFs) and entered in an electronic database. Data monitoring and quality control procedures will be implemented to ensure data accuracy and integrity. Statistical analyses will be performed using appropriate methods, and the results will be reported in accordance with the CONSORT guidelines for randomized controlled trials.

### Public disclosure and publication policy

The trial has been registered at ClinicalTrials.gov on 15/11/2024 before inclusion of the first patient (NCT06690528). The results of the trial will be submitted and published, preferably in high-impact peer-reviewed journal, regardless of the study outcome. The first, second, and last authorships are for the principal investigators and coordinators. For other authorships the ICMJE guidelines will be followed. The criteria for authorship are defined as (1) substantial contributions to conception and design, acquisition of data, or analysis and interpretation of data; (2) drafting the article or revising it critically for important intellectual content; and (3) final approval of the version to be published. Authors should meet conditions 1, 2, and 3.

The SONAR trial is a multicenter trial. For the collaborating centers the following applies: there is a minimum of 3 randomized patients to obtain 1 co-authorship, a minimum of 6 randomized patients for 2 co-authorships and 10 randomized patients for 3 co-authorships. Per site it is internally determined which local investigator(s) will be co-author, primary contact for this process is the local principal investigator. Principal investigators will be mentioned as such in the manuscript. All other authors will be listed in alphabetical order. Clinicians who are involved in this study and do not fulfill the previously mentioned criteria, will be noted as ‘collaborator’ in the final manuscript and the medical journal will be asked to list the names of all collaborators as such in PubMed.

## Discussion

The standard treatment for metastatic pancreatic ductal adenocarcinoma (mPDAC) remains systemic chemotherapy, with the choice of regimen based on the patient’s performance status and overall condition. Current guidelines recommend FOLFIRINOX or gemcitabine plus nab-paclitaxel for patients with good performance status, as these regimens have demonstrated significant survival benefits [23–25]. Surgical resection is generally not considered as a curative option for mPDAC due to the historically poor outcomes associated with metastatic disease. However, growing evidence suggests that in highly selected patients—particularly those with oligometastatic disease confined to the liver and a favorable response to chemotherapy—surgery may provide a survival advantage [26–28]. The identification of suitable candidates is crucial, with key selection criteria including a limited number of liver metastases, a good response to systemic therapy, and the absence of aggressive tumor biology. A complete pathological response of metastases after chemotherapy has emerged as a strong predictor of benefit from surgery, correlating with significantly prolonged survival [18]. Other favorable prognostic factors include low preoperative CA19-9 levels, absence of vascular invasion, and good nutritional and inflammatory status [28, 29]. Retrospective studies indicate that selected patients undergoing resection may achieve a notable survival benefit. Those who attain ypM0 status after chemotherapy have demonstrated median OS of 25.5 months, compared to 10.7 months in patients with residual metastatic disease (ypM1) and 8.1 months in non-resected individuals [18]. Furthermore, multimodal treatment strategies combining surgery with chemotherapy have shown median survival times exceeding 20 months in selected cases [26–28]. Importantly, postoperative morbidity appears to be manageable at high-volume centers with expertise in both pancreas and liver surgery, with relatively low rates of severe complications and no mortality reported in some series [28].

The METAPANC trial [30] is a notable ongoing randomized, controlled phase III study evaluating the role of multimodal therapy, including surgery, in patients with oligometastatic pancreatic cancer. This trial is investigating whether resection of both the primary tumor and liver metastases, in combination with perioperative chemotherapy, can improve survival outcomes. As the first large-scale, international trial on this topic in Western countries, METAPANC represents a crucial effort to establish evidence-based treatment recommendations.

The SONAR trial aims as well to contribute to this growing body of research by rigorously assessing the impact of surgical resection compared to observation or continuation of chemotherapy in a well-defined patient population. By employing a randomized design with strict eligibility criteria, this study will provide valuable insights into the role of surgery in oligometastatic pancreatic cancer. Despite its relatively small sample size, the trial’s robust methodology strengthens its potential impact.

If successful, the SONAR trial could help redefine treatment strategies for select patients with limited metastatic disease, demonstrating the feasibility and clinical benefit of an aggressive multimodal approach. The findings will not only contribute to the evolving landscape of pancreatic cancer management but also serve as a foundation for future research aimed at developing personalized treatment strategies for this challenging disease.

## Data Availability

No datasets were generated or analyzed during the current study.

## Abbreviations

2y-OS: Two-year Overall Survival
AE: Adverse Event
ASCO: American Society of Clinical Oncology
BRCA: Breast Cancer Gene (BRCA1/BRCA2)
Ca 19 − 9: Carbohydrate Antigen 19 − 9
CONSORT: Consolidated Standards of Reporting Trials
CRFs: Case Report Forms
ctDNA: Circulating Tumor DNA
CT: Computed Tomography
DNA: Deoxyribonucleic Acid
ECOG: Eastern Cooperative Oncology Group
EORTC: European Organisation for Research and Treatment of Cancer
EORTC QLQ-C30: European Organisation for Research and Treatment of Cancer Quality of Life Questionnaire-Core 30
EORTC QLQ-PAN26: European Organisation for Research and Treatment of Cancer Quality of Life Questionnaire-Pancreatic Cancer Module
FDG: Fluorodeoxyglucose
FOLFIRINOX: Leucovorin, Fluorouracil, Irinotecan, and Oxaliplatin
GemNabP: Gemcitabine plus Nab-Paclitaxel
IDMC: Independent Data Monitoring Committee
ICMJE: International Committee of Medical Journal Editors
IV: Intravenous
mFOLFIRINOX: Modified FOLFIRINOX
mPDAC: Metastatic Pancreatic Ductal Adenocarcinoma
MRI: Magnetic Resonance Imaging
NCCN: National Comprehensive Cancer Network
N/L Ratio: Neutrophil-to-Lymphocyte Ratio
OS: Overall Survival
PAXG: Cisplatin, Nab-Paclitaxel, Capecitabine, and Gemcitabine
PET: Positron Emission Tomography
PFS: Progression-Free Survival
PS: Performance Status
QoL: Quality of Life
RECIST: Response Evaluation Criteria in Solid Tumors
SONAR: Surgery in Oligometastatic paNcreatic cAnceR
